# Help Others and Yourself Eventually: Exploring the Relationship between Help-Giving and Employee Creativity under the Model of Perspective Taking

**DOI:** 10.3389/fpsyg.2017.01030

**Published:** 2017-06-23

**Authors:** Si Li, Shudi Liao

**Affiliations:** ^1^School of Management, Huazhong University of Science and TechnologyWuhan, China; ^2^School of Business, Hubei UniversityWuhan, China

**Keywords:** creativity, help-giving, organization-based self-esteem (OBSE), organizational citizenship behavior (OCB), perspective taking

## Abstract

Although a plethora of studies have examined the antecedents of creativity, empirical studies exploring the role of individual behaviors in relation to creativity are relatively scarce. Drawing on the model of perspective taking, this study examines the relationship between help-giving during creative problem solving process and employee creativity. Specifically, we test perspective taking as an explanatory mechanism and propose organization-based self-esteem as the moderator. In a sample collected from a field survey of 247 supervisor-subordinate dyads from 2 large organizations in China at 3 time points, we find that help-giving during creative problem solving process positively related with perspective taking; perspective taking positively related with employees’ creativity; employees’ organization-based self-esteem strengthened the link between perspective taking and creativity; besides, there existed a moderated mediation effect. We conclude this paper with discussions on the implications for theory, research, and practice.

## Introduction

Past decades have witnessed the burgeoning research interest in identifying drivers that might facilitate employees’ engagement in creativity, which is defined as the generation of novel and useful ideas (Amabile, 1983; Shalley, 1991). Though studies exploring the antecedents of creativity have accumulated rapidly, most of them are focused on the individual factors like cognition, affect and motivation, and contextual predictors (Shalley et al., 2004). It is surprising, however, that scant literature has considered individual behaviors (e.g., De Stobbeleir et al., 2011; Černe et al., 2014, for exception). As interpersonal process initiated by individuals have a great influence on individual-level creativity (Perry-Smith and Shalley, 2003; Shalley et al., 2004; George, 2007), and in response to Zhou and Hoever’s (2014) call for incorporating new perspectives, this study goes beyond usual practice and discusses individual behavior as predictors of creativity.

Help-giving is one of such a behavior, depicting individuals’ “willing devotion of time and attention to assist with the work of others” (Hargadon and Bechky, 2006, p. 489). During organizations’ progress to achieve creativity, work starts to get more and more complex and interdependent, which makes team or group structures quite pertinent and as a result, team or group structures are becoming more and more prevalent (Stevens and Campion, 1994; Cohen and Bailey, 1997). In this background, to attain group effectiveness, interpersonal facilitation becomes indispensable, of which helping behaviors is one of the most salient forms that could cultivate interpersonal coordination and integration (Van Scotter and Motowidlo, 1996). For example, Hargadon and Bechky (2006) found that employees within organizations implicitly assumed that it is inevitable to ask for help to implement organizational knowledge. Moreover, Perlow (1999) discovered that within the R&D group, individuals have to get help from others to accomplish their work. To sum up, both the empirical and theoretical evidence suggested that helping behaviors are essential and indispensable within organizational progress.

Nonetheless, recent work by Mueller and Kamdar (2011) suggested a negative link between help-giving behavior and creativity. According to their study, help-giving is a “curse” for individual creativity. One of their major arguments is that individuals’ personal resources are finite and limited (Hobfoll, 1989). While, the act of help-giving can diminish the amount of time or energy that could have been devoted to his or her own tasks (Bergeron, 2007). Consistent with this perspective, a wide array of research indicate that helping behaviors positively relate to citizenship fatigue (Bolino et al., 2015), role overload, job stress, work-family conflict (Bolino and Turnley, 2005), and diminish task performance (Bergeron, 2007; Barnes et al., 2008). To extend this logic further, it is easy to assume that help-giving might be harmful for individual creativity as well.

A somewhat thorough perusal of the help-giving literature, however, cast doubt on the assumed negative relationship between help-giving and creativity. Katz and Kahn (1978), for instance, once posited that OCB itself is a spontaneous, and “innovative” behavior. Aligning with this argument, Kesen (2016) found that four dimensions of OCB (conscientiousness, helping, civic virtue and sportsmanship) all positively relate to creativity. The mixed results posit an intriguing question about the actual relationship between help-giving and creativity. Accordingly, instead of denying the existing evidence suggesting its detrimental effects, the present paper aims to explore the potential benefits of help-giving by examining how and when help-giving would benefit employee’s creativity.

According to the model of perspective taking, job-related factors and individual factors both could predict individuals’ perspective taking, thus further results in cooperation-oriented contextual performance (Parker and Axtell, 2001). Drawing upon this model, we propose that help-giving during creative problem solving process might evoke employees’ perspective taking, thus further facilitates their creativity, and this effect is contingent on employee’s organization-based self-esteem (OBSE). Unlike cooperative contextual performance, creativity has always been viewed as a process full of uncertainties and high rates of failure (Simonton, 1984; Metcalfe and Wiebe, 1987; Fleming, 2001). Thus, individual characteristics like self-esteem could play an important role in explaining individuals’ different reactions to the same situations (Brockner, 1988), and here we adopt individuals’ OBSE as the moderator. In doing so, we make several contributions. Firstly, our study complements the current creativity literature, as previous research concerning predictors of creativity predominantly focus on individual difference and psychological process. In response to the call for more attention beyond the previous theoretical perspective (Zhou and Hoever, 2014), the present study finds out that help-giving, as a prosocial behavior, could also contribute to individuals’ creativity. Secondly, based on the model of perspective taking, we disentangle the indirect effect of perspective taking on the relationship between help-giving behaviors and creativity. As extant research concerning the link between helping behaviors and creativity shows inconsistent results, our study argues that workplace interaction such as help-giving could evoke individual’ perspective taking, which provides a novel insight into the mixed link. Finally, our study unearths that employees’ OBSE moderates the link between perspective taking and creativity. While prior research suggests that OBSE directly impacts employee creativity (Chen and Aryee, 2007), our study further points out that it also enhances the positive relationship between perspective taking and creativity. Therefore, we demonstrate the importance to theory and practice of considering self-concept not only as mediators to creativity but also as moderators.

### Theories and Hypotheses

According to the model of perspective taking, Parker and Axtell (2001) mainly adopt two approaches to conceptualize perspective taking. One of which takes on the dispositional approach and considers it as a relatively stable trait depicting individuals’ ability to perceive others’ feelings; the other considers it in a situational approach which suggests that individuals’ perspective taking responds to external factors. Align with Parker and Axtell (2001) as well as our research intention, we are primarily concerned with the situational approach in the present paper. Specifically, perspective taking is a cognitive-affective experience that varies from situations, which suggests that it not only comprises cognitive process, which is referred to as “intellectual empathy,” but also includes affective component, which is referred to as “empathic emotions” (Duan and Hill, 1996). What’s more, based on Duan and Hill’s (1996), the model posits that the cognitive process could result in the affective response of empathy along with positive attributions toward the target. In addition, Parker and Axtell (2001) delineate that job-related attributes like interaction with targets and job autonomy, and individual factors like experience of targets’ job, flexible role orientation and integrated understanding, all could predict perspective taking. Besides, job-related attributes could also influence perspective taking indirectly via individual factors (flexible role orientation and integrated understanding).

Moreover, the model suggests that individuals’ perspective taking could promote contextual performance, especially the cooperation-oriented ones. Notebly, a series of studies have testified and supported the positive link between perspective taking and helping behaviors (c.f., Underwood and Moore, 1982; Oswald, 1996; Batson, 1997; Maner et al., 2002). Nonetheless, with empirical evidence suggesting that there are reliable associations between perspective taking and helping behavior (Underwood and Moore, 1982; Maner et al., 2002), we boldly assume that there might exist a reciprocal process, as indicated by Parker and Axtell (2001) as well. And that is, individuals’ help-giving behaviors could also promote perspective taking, especially during the creative problem solving process.

In the team contexts, help-giving behaviors during creative problem solving process underlies that individuals give help to others by gathering and utilizing information to generate creative outcomes (Mueller and Kamdar, 2011). Specifically, the team contexts indicate the interdependence of the working procedure and their common team goal. In this setting, based upon the model of perspective taking, we propose that individuals’ help-giving during creative problem solving process could evoke their perspective taking behaviors. On the one hand, as part of organizational citizenship behavior (OCB), giving help inevitably initiates social interactions with others, which enables individuals to expose to alternative ways of thinking of the targets that they are giving help, thus further predict perspective taking directly; on the other hand, this kind of social interaction could facilitate integrated understanding and flexible role orientation, thus enhances perspective taking indirectly.

For one thing, help-giving could facilitate integrated understanding. In order to better help those who seek help from them, individuals have to think in their shoes, which could help broaden their perspective about the work context, like how their work relates to each other, and help them have a big picture of the whole woke design.

For another, help-giving behaviors during creative problem solving process could lead to their flexible role orientation in two ways. Flexible role orientation refers to individuals’ emergent and flexible view of their role (Parker et al., 1997). Firstly, one of the salient characteristics of current organizations is the clear-cut division of labor, which means that employees are being assigned with different work tasks and responsibilities, even in the team contexts (Durkheim, 2014). As a result, employees are fully aware of their working boundaries, that is, what is their job and what is not. As help-giving behaviors are out role performance, which means that individuals who exhibit that should exert extra cognitive and emotional effort. Individuals who frequently conduct these behaviors, the boundaries of their job and role expectation could be expanded to include both the self and organizational role (Perlow, 1998; Tepper et al., 2001; Bolino and Turnley, 2005). Accordingly, an emergent and flexible view of their roles might emerge, which means that they will have a broader perspective and a wider acceptance of their job responsibilities. Secondly, help-giving during creative problem solving process usually involves making decisions about different creative alternatives. According to Davis and Wacker (1987), participation in decisions about change make individuals more likely to develop ownership for that change. This ownership for change during the creative problem solving process usually involves accountability for the creative outcomes, rather than merely for its own working outcomes, which refers to flexible role orientation as well. Altogether, individuals are more likely to feel concerned about and accountable for others’ work and their problems and put effort to resolve them, one of the manifestations is perspective taking.

In addition, help-giving behavior might also lead to individuals’ experience of others’ job. Specifically, in certain situations, in order to help others to accomplish their work, individuals need to help others to complete their job or even doing their job for them, which inevitably involves experience of their job, this shared experience of the same events with others creates empathy and perspective taking (Batson et al., 1996).

To sum up, individuals’ help-giving during the creative process could help them to experience others’ job, have a flexible role orientation and an integrated understanding of the workplace. According to the model of perspective taking, these help-giving behaviors could lead to their perspective taking. Thus. We propose that:

**Hypothesis 1:** Help-giving during creative problem solving positively relates to perspective taking.

Meanwhile, the model of perspective taking articulates that perspective taking behaviors could enhance interpersonal facilitation, one of which is cooperative contextual performance that supports the work context. Here, we extend this model and suggest that individuals’ perspective taking provoked by help-giving during creative process could also lead to creativity, which is one of the 10 dimensions of overall job performance (Ng and Feldman, 2008), based on the following two reasons.

As noted before, creativity comprises two orthogonal dimensions: novelty and usefulness (Amabile, 1996). The novelty dimension denotes something new and original, and the usefulness dimension illustrates that creativity also entails utility which suggests that creative ideas need to be useful and valuable to others. While, perspective taking behaviors during the creative process could somehow fulfill both dimensions.

On the one hand, when employees take others’ perspectives selectively, they are spontaneously exposed to various opinions and diverse viewpoints, which could be seen as a prerequisite for creativity to happen since it provides the basic resource, i.e., information, especially during the creative process, peers’ viewpoints are not only relevant but also novel, original, and unique for employees to develop creative ideas. Evidence has suggested that perspective taking behavior can directly contribute to creativity by exposing focal ones to new ideas (Galinsky et al., 2008).

On the other hand, the model of perspective taking demonstrates two fundamental manifestations. One is empathy with the targets, which means individuals will be more likely to identify, understand, and feel concerned about the targets’ experience; the other is positive attributions about the targets, which indicates that attributions about target’s behavior and outcomes are more “self-like” and positive. Both of these two, according to Davis et al. (1996), provide a favored status for the target, whose perspectives have been taken. Besides, Batson and Powell (2003) suggest that perspective taking would inspire altruism. What’s more, Mohrman et al. (2001) suggest that perspective taking between relevant communities, such as researchers and practitioners, could make the associated outcomes like organizational research more useful. To extend this logic further, perspective taking of others during creative process would increase the usefulness of those creative ideas. Taken together, perspective taking could promote individuals who take others’ perspectives to develop ideas that are useful to others.

To sum up, organizational creativity entails both novelty and usefulness (Guilford, 1967). For one thing, the exposure to others’ opinions during the creative process could imperceptibly enhance the novelty of individuals’ own creative idea. For another, the inclusion of others in one’s self-concept could also facilitate the usefulness of the creative ideas. Thus, we propose that:

**Hypothesis 2:** Perspective taking positively relates to creativity.

Hence, we further propose that help-giving behavior during creative problem solving process could contribute to employees’ creativity via the indirect effect of perspective taking. Specifically:

**Hypothesis 3:** Help-giving during creative problem solving process positively relates with creativity via the indirect effect of perspective taking.

According to Brockner (1988), individual difference like self-esteem plays a vital part in individuals’ reactions to external environment. As a situational self-esteem, OBSE refers to the extent to which individuals believe themselves to be worthy, capable and significant as an organization member (Pierce et al., 1989). Notebly, OBSE reflects individual’s self-esteem within organizational contexts and it is construed by individuals’ past direct and personal experience, like task accomplishments and failures (Korman, 1970, 1976; Brockner, 1988). Additionally, Pierce et al. (1989) also indicate that OBSE is better than global self-esteem in predicting organizational-related behaviors and attitudes. Specifically, individuals develop attitudes and engage in behaviors that align with their organizational roles to maintain or enhance their self-esteem. Meanwhile, research has found that OBSE is positively related to a wide range of outcomes, such as job satisfaction, intrinsic motivation, citizenship behaviors and organization-related attitudes and behaviors, etc. (Pierce et al., 1989; Pierce and Gardner, 2004).

Here, we argue that individuals’ OBSE could strengthen the relationship between employees’ perspective taking and their creativity for the following reasoning. According to Korman’s (1970) self-consistency theory, individuals will engage in activities that are consistent with their self-cognition and self-images in job situations. Specifically, individuals with positive images and cognition of themselves would engage in behaviors that enhance that positive image and cognition; whereas, individuals with negative image or cognition would engage in behaviors that are in accordance with the negative image or cognition. To extend this logic further, unlike cooperation-oriented contextual performance, scholars have suggested that the creative process are often accompanied by uncertainties and unpredictability as well as high rates of failure (Simonton, 1984; Metcalfe and Wiebe, 1987; Fleming, 2001). Thus, individuals with low OBSE might be uncertain and lack of confidence of their capability to overcome barriers and pursue creative outcomes, thus avoid the creative endeavor. In contrast, as construed by their past experience of success and failure, individuals with high OBSE usually view themselves as valuable, competent and meaningful existence within the organizations, thus are more likely to conduct behaviors that could exhibit their competence and boost their organizational contribution. In the context of the creative team process, thinking out a creative idea could definitely demonstrate one’s personal competence and elevate one’s standing within teams, thus individuals with high OBSE might persist on the pursuit of creativity regardless of the uncertainties and possible failures. Research has echoed this argument by demonstrating that even accompanied by creative self-efficacy and creativity skills, the lack of basic skill or general efficacy could also prevent the occurrence of creativity (Amabile, 1983, 1988; Tierney and Farmer, 2002). When individuals lack confidence of their basic capability to conduct job within teams, even in the presence of strong creativity resources, it is still hard for them to utilize those creativity resources and generate creative outcomes. Hence:

**Hypothesis 4:** Organization-based self-esteem moderates the relationship between perspective taking and creativity such that the positive relationship between perspective taking and creativity strengthens when OBSE is higher.

As was stated above, individuals’ OBSE moderates the link between perspective taking and creativity, it is also possible that individuals’ OBSE can influence the strength of the indirect effect of help-giving behaviors during creative problem solving process and creativity. Hence, we propose that:

**Hypothesis 5:** Organization-based self-esteem moderates the positive indirect effect of help-giving during creative problem solving and creativity (through perspective taking). Specifically, higher levels of OBSE strengthens the extent to which perspective taking mediates the indirect effect of help-giving during creative problem solving and creativity.

**FIGURE 1:**
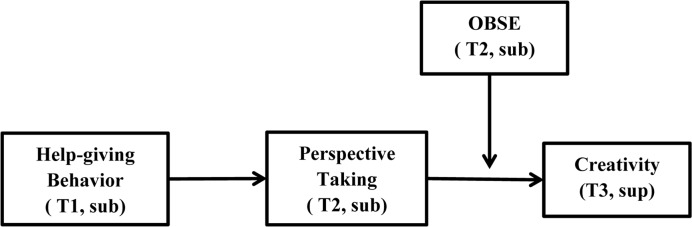
Theoretical framework. Notes: Help-giving behavior: Time 1 by subordinate; Perspective taking: Time 2 by subordinate; OBSE: Time 2 by subordinate; Creativity: Time 3 by supervisor.

**Figure 1** summarizes our proposed theoretical model.

## Materials and Methods

### Sample and Procedure

We collected data from two large logistics enterprises distributed in two cities, including Guangzhou, and Shanghai, in China within 3 months. The research departments of both the two companies mainly comprised project teams who are responsible for the planning of logistics routes and coping with all kinds of emergency, which entails rapid response capabilities and creative problem solving capabilities. To avoid common method bias, we adopt a three-wave study, and the interval between each investigation was 2 months. Employees completed their questionnaires in the first and second phases, then their corresponding supervisors finished their questionnaires in the third phase. All the employees and supervisors completed their questionnaires on their own without discussion. Then we collected all the on-site questionnaires immediately. The operations of all the investigations were the same. The surveys were originally constructed in English and then translated into Chinese using the back-translation procedure for the assurance of equivalence of the measures (Brislin, 1986).

During the first investigation, we distributed 450 dyadic questionnaires and retrieved 327 dyadic questionnaires, and the response rate was 72.67%; the second investigation then targeted at these 327 dyads and retrieved 283 dyadic complete questionnaires, and the response rate was 86.54%; the third investigation then targeted at these 283 dyads, and we collected 247 valid dyadic questionnaires, and the response rate was 87.28%. Among the supervisors, male supervisors account 46.4%, the average age is 38.35, the average position tenure is 7.2 years, 67.9% of them have bachelor’s degree and higher qualifications. In the samples of employees, 42.9% are male employees, the average age is 31.88, 65.2% of them have bachelor’s degree and higher qualifications, average position tenure is 5.4 years, average organization tenure is 7.42 years.

### Ethics Statement

Before starting the data collection, we consulted our university’s ethics committee and was approved to conduct this research. According to our research design, the study did not violate any legal regulations or common ethical guidelines. To further ensure the ethical innocuousness of the study, before distributing questionnaires, we again introduced our research goal, and the research plan concerning each participants to them, and asked for their permission and consent to participate in the investigations, and emphasized that as soon as they feel inappropriate, they could quit and their incomplete data would be deleted and not assessed.

### Measures

Unless otherwise indicated, all items used a 7-point scale, ranging from “strongly disagree”(1) to “strongly agree”(7).

#### Help-Giving Behavior (Time 1)

We adapted a six-item scale from Mueller and Kamdar (2011) which focuses on specific help-giving behaviors targeting creative problem solving process. Employees self-report their help-giving behaviors at the first stage. Sample items include “I go out of my way to help coworkers refine their creative ideas”(Cronbach’s alpha = 0.923).

#### Perspective Taking (Time 2)

Employees indicated their extent to take others’ views into consideration with a four-item scale developed by Davis et al. (1996). Sample item is “On the job, I frequently try to take other people’s perspectives” (Cronbach’s alpha = 0.946).

#### Organization-Based Self-esteem (Time 2)

Employees reported their OBSE using a 10-item scale developed by Pierce et al. (1989). Sample items include “I can make a difference.” (Cronbach’s alpha = 0.911).

#### Creativity (Time 3)

Supervisors rated employee’s creativity using 13-items scale developed by Zhou and George (2001). Sample items include “Comes up with new and practical ideas to improve performance.”(Cronbach’s alpha = 0.973).

#### Control Variables

Employees’ demographic variables have been controlled, such as age, gender, educational levels, and organization tenure, because of their potential effects on creativity (Mumford and Gustafson, 1988; Mumford, 2003; Shin and Zhou, 2003); Moreover, we also controlled the organizational membership to remove any possible biases by adopting dummy-coded variables.

## Results

We then conducted confirmatory factor analyses with maximum likelihood estimation to examine the distinctness of the variables.

### Confirmatory Factor Analysis Results

As indicated in **Table 1**, confirmatory factor analyses indicated that the hypothesized factor structure fitted these data better than the alternative models examined (e.g., χ^2^(481) = 1336.548, *p* < 0.001, RMSEA = 0.085, CFI = 0.902, IFI = 0.903, χ^2^/*df* = 2.779). Moreover, we conducted a more rigorous test to assess the discriminant validity (Hair et al., 2010). By comparing the average variance extracted values for any of the two constructs with the square of the correlation estimate between these two constructs, we could assess the discriminant validity. And, we calculated the AVES (average variance extracted) of each construct and they are all above 0.5, and are greater than the squared correlation estimate. Moreover, the AVE values of any two constructs are also greater than their squared correlation estimate. In addition, we also calculated the composite reliability of each construct, which is above 0.9. See **Table 2**.

**Table 1:** Summary of model fit indexes.

Factor structure	*x*^2^	*df*	*x^2^/df*	IFI	CFI	RMSEA
One-factor model: (Help-giving + Perspective taking + OBSE + Creativity)	4438.252	487	9.113	0.550	0.548	0.182
Two-factor model: (Help-giving + Perspective taking + OBSE), Creativity	2922.524	486	6.013	0.722	0.721	6.013
Three-factor model: Help-giving, (Perspective taking + OBSE), Creativity	2045.643	484	4.227	0.822	0.821	4.227
Four-factor model: Help-giving, Perspective taking, OBSE, Creativity	**1336.548**	**481**	**2.779**	**0.903**	**0.902**	**0.085**

*Values in bold indicate the best-fitting model. According to one of the reviewers, we delete the item of which its factor loading is below 0.5, the fit indexes of the four-factor model are x^2^ = 1224.136, df = 451, x^2^/df = 2.714, IFI = 0.910, CFI = 0.910, RMSEA = 0.084.*

**Table 2:** Reliability and discriminant validity.

Variable	Cronbach’s alpha	AVE (average variance extracted)	Composite reliability
Help-giving behavior	0.923	0.675	0.925
Perspective taking	0.946	0.814	0.946
OBSE	0.911	0.528	0.913
Creativity	0.973	0.738	0.973

	**Shared variance (square of correlation estimate)**		
	**Help-giving behavior**	**Perspective taking**	**OBSE**

Help-giving behavior	1		
Perspective taking	0.095	1	
OBSE	0.085	0.263	1
Creativity	0	0.027	0.014

### Descriptive Statistics and Correlations

**Table 3** shows the descriptive statistics, intercorrelations, and reliabilities for the study variables. Organization tenure negatively relates with individuals’ help-giving behaviors (β = -0.197, *p* < 0.01), but positively relates with individuals’ creativity (β = 0.213, *p* < 0.01). Help-giving behavior positively relates with perspective taking (β = 0.309, *p* < 0.01), perspective taking positively relates with OBSE (β = 0.513, *p* < 0.01), and creativity (β = 0.163, *p* < 0.01). And, OBSE positively relates with individuals’ creativity (β = 0.119, *p* < 0.05).

**Table 3:** Means, standard deviations (SD), and correlations^a^.

Variable	Mean	*SD*	1	2	3	4	5	6	7
(1) Gender	1.543	0.499	1						
(2) Age	31.933	7.548	-0.138ˆ*	1					
(3) Education	2.671	0.704	-0.135ˆ*	0.047	1				
(4) Org. Tenure	7.154	7.059	-0.079	0.788ˆ**	-0.007	1			
(5) Help-giving behavior	5.271	0.745	-0.007	-0.102	0.063	-0.197ˆ**	**–**		
(6) Perspective taking	5.715	0.571	-0.102	-0.002	0.004	0.012	0.309ˆ**	**–**	
(7) OBSE	4.734	0.686	-0.078	-0.051	0.128ˆ*	-0.056	0.291ˆ**	0.513ˆ**	**–**
(8) Creativity	5.184	0.742	-0.125	0.173ˆ**	0.082	0.213ˆ**	0.017	0.163ˆ**	0.119ˆ*

*^a^ n = 247; ^∗^ p < 0.05; ^∗∗^ p < 0.01; ^∗∗∗^ p < 0.001.*

### Results of Tests of the Hypotheses

Since undertaking mediation analysis, Baron and Kenny’s (1986) approach is no longer considered appropriate, to further confirm the hypotheses and test the moderated mediation effect, we adopt Hayes’s PROCESS Macro for SPSS to measure the indirect effects for mediation and moderated mediation (Hayes, 2013), which is also recommended by Edwards and Lambert (2007). The results are reported in **Table 4**. By using 5,000 bootstrap estimates for the construction of 95% bias-corrected CIs, the results demonstrated that the direct effect of help-giving behavior on employees’ creativity was not significant, as the confidence intervals (CI) included zero (95% CI [-0.119,0.142]). While, help-giving behavior was significantly positively related to perspective taking (β = 0.295, *p* < 0.001, 95% CI [0.193,0.396]), and perspective taking was significantly and positively related to creativity as well (β = 0.205, *p* < 0.05, 95% CI [0.028,0.382]). Thus, hypothesis 1 and 2 were supported. Additionally, the interaction between perspective taking and OBSE proved to have a significantly positive effect on creativity (β = 0.230, *p* < 0.05, 95% CI [0.052,0.408]). Hypothesis 4 was supported. Moreover, the index of the moderated mediation effect was 0.068, the CI (0.017,0.145) does not include zero, which suggests that employees’ OBSE moderates the indirect effect of help-giving during creative problem solving and creativity (through perspective taking). Thus, hypothesis 5 was supported.

**Table 4:** Bootstrap confidence intervals for the hypothesized moderated mediation effects.

Variable	Perspective taking			Creativity		
	Coefficient	*SE*	95% CI	Coefficient	*SE*	95% CI
*Control variables*						
Company	-0.024	0.039	-0.100,0.053	-0.348ˆ***	0.046	-0.439,-0.256
Gender	-0.120	0.077	-0.273,0.032	-0.016	0.093	-0.199,0.166
Age	-0.007	0.009	-0.024,0.010	0.009	0.010	-0.012,0.029
Education	-0.039	0.059	-0.155,0.078	-0.134	0.071	-0.274,0.007
Org. Tenure	0.010	0.009	-0.009,0.028	-0.010	0.011	-0.032,0.012
*predictor*						
Help-giving behavior (T1)	0.295ˆ***	0.051	0.193,0.396	0.011	0.066	-0.119,0.142
Perspective Taking (T2)				0.205ˆ*	0.090	0.028,0.382
*OBSE(T2)*				-0.001	0.075	-0.148,0.147
*Interactions:* Perspective Taking × OBSE				0.230ˆ*	0.090	0.052,0.408
Constant	-1.050ˆ*	0.413	-1.864,-0.236	6.062ˆ***	0.505	5.068, 7.056
***R^2^***	*R*^2^ *= 0.132*			*R*^2^ *= 0.278*		
***F***	*F*(6,238) = 6.032 *p* < 0.001			*F*(9,235) = 10.038 *p* < 0.001		

*^∗^ p < 0.05; ^∗∗^ p < 0.01; ^∗∗∗^ p < 0.001.*

The results for hypothesis 3 and 5 are reported in **Table 5**. Results from the bootstrapped data showed that at 1 standard deviation above the mean on our moderator variable (OBSE), the conditional indirect effect was significant (β = 0.111, 95% CI [0.039,0.212]). In contrast, as 1 standard deviation below the mean, the conditional indirect effect was small (β = 0.010), and the 95% CI does contain zero [-0.049,0.072]).

**Table 5:** Moderated mediated results for perspective taking across levels of organization-based self-esteem (OBSE)^∗^.

	Perspective taking			
Moderator = OBSE	Conditional indirect effect	*SE*	95% BootLLCI	95% BootULCI
Low (-1 SD)	0.010	0.030	-0.049	0.072
High (+1 SD)	0.111	0.043	0.039	0.212
	
	**Bootstrapping effect**	***SE***	**95% CI (LL,UL)**	
	
Moderated mediation effect	0.068	0.032	0.017,0.145	

*^∗^Bootstrap 5,000.*

**FIGURE 2:**
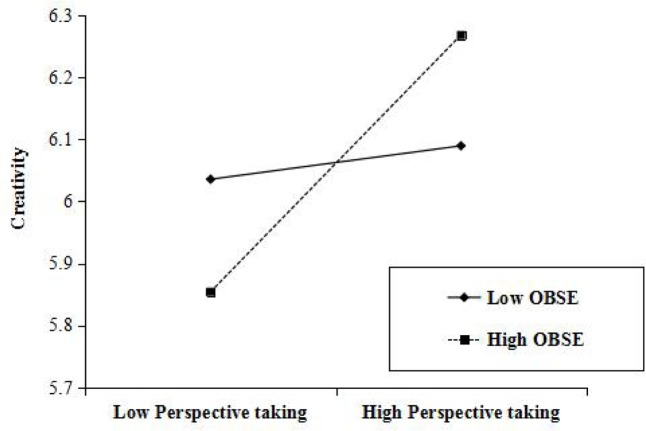
Interaction between employee’s perspective taking and OBSE predicting employee’s creativity.

**Figure 2** presents the moderating effects between variables.

## General Discussion

Drawing upon the model of perspective taking, we investigated the link between help-giving during creative problem solving process and creativity. In particular, we found that perspective taking mediated the relationship between these two, while employees’ OBSE moderated the link between perspective taking and creativity. Furthermore, employees’ OBSE affected the strength of the link between help-giving behavior and creativity via the indirect effect of perspective taking.

### Theoretical Implication

We contribute to the creativity literature by unveiling the behavioral mechanisms that fuel creativity. As previous studies usually focus on the cognitive, affective and motivational predictors of creativity, few have examined individuals’ behaviors. Recent work by Mueller and Kamdar’s (2011) suggests that help-seeking behavior could also facilitate creativity. The present study manifests that help-seeking is not the only interpersonal process that could contribute to creativity, help-giving can also be conducive to creativity via the indirect effect of perspective taking, especially during the creative problem solving process. Meanwhile, in the same work, Mueller and Kamdar (2011) also found that help-giving behaviors initiated by previous help-seeking behaviors negatively relates to creativity. In their work, helping giving behaviors are viewed as reciprocation costs of help-seeking, which further leads to performance costs, mainly by diminishing their amount of time and energy that could be devoted to fulfill their own work and by enhancing their problem representation. To explain the difference, firstly, taken into consideration of the reciprocity of help-giving and help-seeking, the diminished amount of time and energy could be replenished by the reciprocal help-seeking behaviors; secondly, the enhanced problem presentation mainly comes from the notion that individuals who seek help from others acknowledge inferiority to other people and might be viewed as less competent than the help-givers in the given domain (Lee, 2002). However, according to Hargadon and Bechky (2006), seeking help from others could also be perceived as a necessary means to bring organization’s knowledge to bear during the creative problem solving process and several organizations have set up some regulations to reinforce the help-seeking behaviors. Accordingly, giving help would be more likely viewed as regular instead of the help-givers be more competent and superior to the help-seekers. Thus, there could also be a positive link between help-giving behaviors and creativity. The present study testified that employees’ help-giving behavior during creative problem solving process could also contribute to individuals’ creativity via the indirect effect of perspective taking. In doing so, this might offer an explanation why the causal relationship between help-giving behavior and creativity turns out to be uncorrelated, the possible positive and negative effect could neutralize and offset each other ’s influence.

Besides, based upon the model of perspective taking, we find that individuals’ perspective taking behavior could mediate the link between help-giving behavior and their creativity, which add to the literature of discussion on the mixed results of help-giving behaviors. Besides, in doing so, we also enriched the model of perspective taking. For one thing, both the model of perspective taking and a wide array of research concerning the link between perspective taking and helping behaviors all propose that individuals’ perspective taking behaviors could predict their cooperative contextual behaviors, like helping behaviors (Oswald, 1996; Vaish et al., 2009). Our study suggested that this could also be the other way around, that is, help-giving behaviors could also predict individuals’ perspective taking, which provides a new perspective to view the perspective taking model. For another, the model of perspective taking maintains that perspective taking behaviors could promote cooperative contextual behaviors as it enhances interpersonal facilitation. Our study suggested that under certain circumstance, individuals’ perspective taking behavior could also facilitate job performance like creativity, which indicates a virtuous circle that during the creative process, help-giving behaviors among coworkers could further promote focal employees’ creative performance.

We also contribute to the OCB literature by suggesting that help-giving could be beneficial to individual creativity. Recent days, there exists two streams of literature addressing conflicting viewpoints about help-giving behaviors. One is the *enrichment-based* perspective, which suggests that help-giving behaviors positively relate to a number of individual- and organization-level outcomes, such as positive affect (Koopman et al., 2016), task performance (Allen and Rush, 1998; Hoffman et al., 2007), promotion (Hui et al., 2000), decreased unit-level turnover (Podsakoff et al., 2009), and better well-being (Lam et al., 2016). The other is the emerging *depletion-based* perspective, which indicates that help-giving behaviors positively relate to citizenship fatigue, role overload, job stress, work-family conflict, and decreased task performance. This study contributes to the enrichment-based perspective by showing that mediated by perspective taking, employees’ help-giving behavior could also positively contribute to their creativity.

Besides, we also add to the OBSE literature. As research suggests that self-esteem plays a vital part in predicting individuals’ motivation, work-related attitudes and behaviors (Pierce et al., 1989; Pierce and Gardner, 2004; Lee and Peccei, 2007), previous research concerning the link between OBSE and creativity usually focuses on its direct effect, that is, how work characteristics and contextual factors like leadership could affect individuals’ creativity via the mechanism of OBSE (c.f., Cohen-Meitar et al., 2009; Zhang et al., 2015). The present study finds out that as an individual difference, OBSE could also be the boundary condition that moderates the link between perspective taking and creativity. Moreover, about the moderating effect of OBSE, align with the behavioral plasticity theory (Brockner, 1983, 1988), most of the work supported the notion that individuals with low self-esteem are more susceptible and reactive to external influence than individuals with high self-esteem (Pierce et al., 1993; Jex and Elacqua, 1999; Brutus et al., 2000; Hui and Lee, 2000). The present study suggests that this should be divided into two, for the negative external cues, individuals with low self-esteem would be more responsive than individuals with high self-esteem; for positive external cues, individuals with high self-esteem would be more responsive than individuals with low self-esteem (see also, Stark et al., 2000). Thus, we enrich the understanding of research on OBSE.

### Managerial Implications

Firstly, our study suggested that a team-based work context is productive when help-giving behaviors during the creative problem solving process could elicit employees’ perspective taking; secondly, we re-evaluate the value of help-giving, since the depletion-based perspective are becoming more and more pervasive, we are afraid that there will be less and less help-giving behaviors in workplace, which we could also tell from the emerging and intensified knowledge hiding, selfishness in work groups (Margolis, 1984; Černe et al., 2014). Thus, we hope this study might offer some enlightenment and evidence to reassure their concern of giving a hand to others during work. Moreover, organizations should recruit individuals with high OBSE and also cultivate their employees’ OBSE during work in order to enhance their motivation to conduct creative endeavors.

### Limitations and Directions for Future Research

In addition, the present study is not without limitations. Firstly, we did not control the help-seeking behavior, given the importance of this behavior in the discussion of the link between help-giving and creativity, future studies should control it; secondly, we collected data from a field survey, which cannot conclusively rule out alternative directionality. Nonetheless, our research design of collecting data in three-pointed phases and multi-source can increase confidence in the temporal directionality and the generalization of our results. Besides, field design has its own disadvantages, for example, our study just implied variables that we are interested, we can’t rule out other alternatives, so we also call for experiments to replicate our results. Also, we adopt a subject way to measure creativity (i.e., supervisors’ ratings), which could be biased, for example, by the halo effect. Although it is common in the organizational creativity literature to use supervisors as a measure of creativity, it is of significance for further research to use both supervisor ratings and objective measures to reflect employees’ actual creative performance. Moreover, as we collected our data in China, there might be some problems with the generalization of our research findings. However, we collected our research samples in two of the most open cities in China, and most of the business both of the two logistics enterprises are dealing with is foreign trade. More importantly, both of the two logistics enterprises are international companies, within which their employees flow between the international branches. Thus, we can conclude that our research findings could be generalized to other population. Nonetheless, future studies should still replicate our research using different samples to validate our findings. Lastly, we just control the demographics of employees and the company membership, future research could explore other variables which influence the relationship.

## Author Contributions

SiL and ShL contribute to the paper in different ways. And we really appreciate the work of the associate editor and the three reviewers.

## Conflict of Interest Statement

The authors declare that the research was conducted in the absence of any commercial or financial relationships that could be construed as a potential conflict of interest.
