# Supplementary material for: Help Others and Yourself Eventually: Exploring the Relationship between Help-Giving and Employee Creativity under the Model of Perspective Taking
Source: Front Psychol. 2017 Jun 23;8:1030. doi: 10.3389/fpsyg.2017.01030 (PMC5481401; doi:10.3389/fpsyg.2017.01030)
Supplement: Supplementary file 1 [file Data_Sheet_1.docx]

**APPENDIX**

**Scales and Items**

**Perspective taking (**Davis et al., 1996)

1. On the job, I frequently try to take other people’s perspectives
2. At work, I often imagine how other people are feeling
3. On the job, I make an effort to see the world through others’ eyes
4. At work, I regularly seek to understand others’ viewpoints

**Creativity (**Zhou & George, 2001)

1. Suggests new ways to achieve goals or objectives.

2. Comes up with new and practical ideas to improve performance.

3. Searches out new technologies, processes, techniques, and/or product ideas.

4. Suggests new ways to increase quality.

5. Is a good source of creative ideas.

6. Is not afraid to take risks.

7. Promotes and champions ideas to others.

8. Exhibits creativity on the job when given the opportunity to.

9. Develops adequate plans and schedules for the implementation of new ideas.

10. Often has new and innovative ideas.

11. Comes up with creative solutions to problems.

12. Often has a fresh approach to problems.

13. Suggests new ways of performing work tasks.

**OBSE (**Pierce, Gardner, Cummings, & Dunham, 1989)

1. I count around here
2. I am taken seriously around here
3. I am important around here
4. I am trusted around here
5. There is faith in me around here
6. I can make a difference around here
7. I am valuable around here
8. I am helpful around here
9. I am efficient around here
10. I am cooperative around here

**Help-giving behavior during creative process (**Mueller & Kamdar, 2011)

1. I assist coworkers with difficult problem-solving assignments, even when assistance is not directly requested
2. I go out of my way to help coworkers refine their creative ideas
3. I take on extra responsibilities in order to help coworkers solve problems creatively
4. I assist coworkers when they run into problems that are difficult to solve
5. I assist coworkers when they can not think out creative ideas
6. I provide help when coworkers need creative ideas at work, even when that is not my responsibility.

Note: all items are measured by a seven-point scale, ranging from “strongly disagree”(1) to “strongly agree”(7).

**Factor Loading**

| **Items of Construct** | **Factor loading** | **Items of Construct** | **Factor loading** |
| --- | --- | --- | --- |
| OBSE1 | .303 | Creativity1 | .833 |
| OBSE2 | .648 | Creativity2 | .804 |
| OBSE3 | .558 | Creativity3 | .883 |
| OBSE4 | .710 | Creativity4 | .872 |
| OBSE5 | .701 | Creativity5 | .825 |
| OBSE6 | .526 | Creativity6 | .922 |
| OBSE7 | .922 | Creativity7 | .902 |
| OBSE8 | .956 | Creativity8 | .911 |
| OBSE9 | .844 | Creativity9 | .784 |
| OBSE10 | .695 | Creativity10 | .903 |
|  |  | Creativity11 | .816 |
| Help-giving1 | .851 | Creativity12 | .859 |
| Help-giving2 | .900 | Creativity13 | .792 |
| Help-giving3 | .838 | perspective taking1 | .882 |
| Help-giving4 | .868 | perspective taking2 | .913 |
| Help-giving5 | .764 | perspective taking3 | .926 |
| Help-giving6 | .681 | perspective taking4 | .889 |
